# Développement d´un questionnaire d´évaluation des apports calciques journaliers chez le Camerounais (QUEVACC)

**DOI:** 10.11604/pamj.2023.46.1.41310

**Published:** 2023-09-01

**Authors:** Jan René Nkeck, Hélène-Ornella Bongha Ondoa, Saquinatou Hamadjoda, Doris Bibi Essama, Baudelaire Fojo Talongong, Madeleine Ngandeu-Singwe

**Affiliations:** 1 Equipe de Recherche en Rhumatologie de Yaoundé (ERRY), Yaoundé, Cameroun,; 2 Laboratoire de Rhumatologie Clinique, Faculté de Médecine et des Sciences Biomédicales, Université de Yaoundé I, Yaoundé, Cameroun,; 3 Service de Médecine Interne, Centre Hospitalier Régional d´Ebolowa, Ebolowa, Cameroun,; 4 Service de Rhumatologie, Hôpital Central de Yaoundé, Yaoundé, Cameroun

**Keywords:** Calcium intake, Cameroon, QUEVACC

## Aux éditeurs de Pan African Medical Journal

L'évaluation des apports calciques est capitale dans la prévention des maladies osseuses en rapport avec le métabolisme phosphocalcique, telles que l'ostéoporose et l´ostéomalacie, qui représentent actuellement dans le monde un problème de santé publique [1]. Plusieurs outils peu coûteux ont été développés dans le but d´aider le clinicien à estimer en pratique courante les apports en calcium journalier chez le patient reçu en consultation. Parmi eux, les plus utilisés sont l´auto-questionnaire de Fardellone, le questionnaire du CERIN, le questionnaire CoCoNut, et le Calcium Assessment Tool (CAT) [2-5]. Ces outils, pouvant être auto-administrés ou administrés par le clinicien, ils évaluent les apports calciques journaliers à partir des données des aliments les plus consommés dans une population donnée, et dont on connait la teneur en calcium. L'usage d'un questionnaire portant sur l´alimentation est conditionné par les habitudes alimentaires de la population cible. Les différences culturelles au plan culinaire et la diversité alimentaire en Afrique Subsaharienne (ASS) rendent complexe l´applicabilité de ces questionnaires. Ainsi, pour être utilisés en pratique, il faudrait au préalable une adaptation de ces outils, suivie d'une validation au sein de la population concernée. À ce propos, nous proposons l´outil QUEVACC (Questionnaire d´Evaluation des Apports en Calcium chez le Camerounais), Tableau 1, Tableau 1.1, qui est une adaptation du CAT à partir des données locales sur les ressources alimentaires au Cameroun et en ASS, et la teneur des aliments sélectionnés en calcium [6-8].

**Tableau 1:** outils QUEVACC (Questionnaire d’Évaluation des Apports en Calcium chez le Camerounais)

Groupe d’aliments	Apport calcique par aliment	Proportion de l’aliment	Evaluation pratique locale	n	Apport calcique
	Aliments apportant 50mg de Calcium par repas				
Légumes et fruits	Gombo bouilli (okra)	60g	4 gombos moyens		50mg multiplié par la somme
	Dattes séchées	80g	8 dattes		des « n »
	Concentré de tomates	160g	8 cuillères à soupe rases		
	Ananas	250g	1 tranche de 100 FCFA		
	Mangue chair	250g	3 petites mangues		
	Orange mure	250g	4 oranges de 50 FCFA		
	Papaye	250g	2 tranches de 100 FCFA		
Féculents	Pain de blé complet non enrichi	50g	2 tranches		
	Patate douce	60g	1 petit morceau de patate		
	Farine d’igname	75g	8 cuillères à soupe rases		
	Farine de manioc	80g	10 cuillères à soupe rases		
	Manioc blanc râpé et grillé (Gari, tapioca)	120g	12 cuillères à soupe rases		
	Pain de blé non enrichi	150g	3 boules de pains de 50 FCFA		
	Manioc	150g	1 morceau moyen		
	Maïs bouilli	200g	2 petits épis de maïs		
	Riz brun bouilli	250g	13 cuillères à soupe rases		
	Farine de maïs jaune	250g	3 pots de yaourt de 125g		
	Farine de maïs blanc	250g	3 pots de yaourt de 125g		
	Pâte fermentée de farine de manioc (water foufou)	250g	1 boule moyenne		
VVPOLAV	Poisson chat	50g	1 petit morceau		
	Rognon de bœuf bouilli	50g	1/3 rognon		
	Crabe	70g	1 pince de crabe		
	Carpe d’Afrique	100g	1 petit morceau		
	Œufs de poule frits	100g	2 œufs de 75 FCFA (50g)		
	Œufs de poule bouillis	100g	2 œufs de 100 FCFA (75g)		
	Lapin	100g	½ cuisse de lapin		
	Tripes de bœuf (missounga)	100g	3 petites tripes de 100 FCFA		
	Foie de bœuf	100g	4 petits morceaux de 100 FCFA		
	Chèvre (teneur modérée en matières grasses)	100g	4 petits morceaux		
	Maquereau	100g	1 petit morceau		
	Escargots cuits	200g	2 tasses bombées		
	Agneau	200g	8 petits morceaux		
	Mouton	200g	8 petits morceaux		
	Haricots œil noir (koki)	200g	2 grandes louches rases		
	Blanc de poulet	250g	Moitié d’une poitrine de poulet		
	Apport calcique alimentaire par semaine	mg		Apport calcique alimentaire par semaine	mg
	Apport calcique alimentaire par jour	= mg/jour		Apport calcique alimentaire par jour	= mg/jour
	Apport calcique journalier par les suppléments alimentaires	= mg/jour	Apport calcique journalier par les suppléments alimentaires	= mg/jour	Apport calcique journalier par les suppléments alimentaires
	Apport calcique total (toutes les sources)	= mg/jour	Apport calcique total (toutes les sources)	= mg/jour	Apport calcique total (toutes les sources)

**Tableau 1.1:** outils QUEVACC (Questionnaire d’Évaluation des Apports en Calcium chez le Camerounais)

Groupe d’aliments	Apport Calcique par aliment	Proportion de l´aliment	Évaluation pratique locale	n	Apport calcique
Fruits à coques et graines	Pistache africain (Egusi)	50g	1 petite louche bombée		
	Arachide séché cru	110g	1 grande louche		
	Aliments apportant 75mg de Calcium par repas				
Légumes et fruits	Feuilles amères de Vernonia (Ndolè)	40g	1 petite boule égouttée		75mg multiplié par la somme
	Oseille de guinée (foléré)	50g	1 petite boule égouttée		des « n »
	Feuille chou caraïbe (macabo)	100g	2 grandes louches bombées		
VVPOLAV	Soja sec	30g	4 cuillères à soupe		
	Tilapia	60g	1 petit morceau		
	Haricot	70g	3 cuillères à soupe		
	Soja trempé	90g	5 cuillères à soupe		
Fruits à coques et graines	Pate d´arachides	120g	1 pot de yaourt de 125g		
	Aliments avec 150mg de Calcium par repas				
Légumes	Feuilles d´amarante (Folong)	40g	1 petite boule égouttée		
et fruits	Feuilles de manioc (Kwem)	50g	Grande louche bombée		
	Légumes feuilles vert frais	70g	2 petites boules égouttées		
Féculents	Taro tubercule	75g	2 petits morceaux		
Produits	Lait de chèvre frais	100g	1 tasse de café		
laitiers et alternatives végétales	Yaourt de lait de vache partiellement écrémé	100g	1 tasse de café		
	Aliments avec 250mg de Calcium par repas				
Légumes et fruits	Corète potagère (Kèlen kèlen)	20g	Moitié d’un petit bol de 250ml		250mg multiplié par la somme
VVPOLAV	Crevettes entières séchées	20g	Moitié d´une poignée		des « n »
	Sardines à l´huile en conserve, égouttées avec arêtes	60g	½ boite classique		
Produits laitiers et	Lait de vache en poudre écrémé	20g	3 cuillères à soupe		
alternatives végétales	Lait de vache en poudre entier	25g	2 sachets de 100 FCFA (12g)		
	Apport calcique alimentaire par semaine				mg
	Apport calcique alimentaire par jour				= mg/jour
	Apport calcique journalier par les suppléments alimentaires				= mg/jour
	Apport calcique total (toutes les sources)				= mg/jour

Le développement de l´outil QUEVACC a été réalisé en quatre étapes: 1) la constitution d´un comité d´experts comprenant quatre rhumatologues, un endocrinologue et un médecin nutritionniste. 2) le choix d'un questionnaire validé dans la littérature, pouvant prendre en compte une grande diversité alimentaire pour l´estimation des apports en calcium. Ce choix s'étant porté sur le CAT. 3) l'insertion au sein du questionnaire, pour chaque apport considéré en calcium (50mg, 75mg, ou 250mg), les aliments riches en calcium avec la proportion correspondante. Ces aliments ont été sélectionnés dans le référentiel de l´Organisation des Nations Unies pour l´alimentation et l´agriculture basé sur les données de l´Afrique de l´Ouest, en considérant les aliments riches en calcium (teneur moyenne ≥ 20mg de calcium pour 100g d'aliment), parmi les plus consommés au sein de la population camerounaise [6]. Pour une meilleure évaluation de la quantité hebdomadaire de l´aliment ingéré, nous avons suggéré une adaptation pratique locale. 4) L'outil ainsi développé a été testé en milieu semi urbain, auprès de 50 participants volontaires âgés de 30 à 60 ans vus en consultations externes de rhumatologie au Centre Hospitalier Régional d´Ebolowa (Région du Sud, Cameroun) pendant le mois de juillet 2023. Les participants ayant pris part avaient tous une bonne compréhension de la langue française avec un niveau scolaire au moins secondaire, ne nécessitant pas de traduction en langage local. Nous n´avons pas inclus les personnes malvoyantes ou ayant un problème neuropsychiatrique. Nous avons relevé un temps médian de réponses de 12 minutes [Quartiles 25-75: 10-19], un recours à l´aide par le participant et son entourage chez 34/50 participants.

L'outil QUEVACC, tout comme le CAT, permet d'évaluer la consommation hebdomadaire et journalière des aliments riches en calcium le mois précédant l´entrevue avec le praticien. Les aliments consommés régulièrement, mais à une fréquence estimée à moins d´une fois par semaine sur le mois précédent, ne sont pas comptés dans les apports calciques hebdomadaires et journaliers. Il s'agit du premier questionnaire estimant en pratique les apports calciques chez le Camerounais, et l´un des rares en ASS. Le Cameroun étant un micro écosystème d´Afrique subsaharienne, ce questionnaire peut être évalué et adapté dans d´autres sous-groupes d´ASS avec des cultures alimentaires similaires. Cependant, il advient de réaliser une validation convenable du questionnaire sur une population large et de développer une version simplifiée qui pourra être auto administrée ou exploitée par les personnes ayant un niveau scolaire bas, et éventuellement des versions en langage local.
